# Characteristics of global retractions of schizophrenia-related publications: A bibliometric analysis

**DOI:** 10.3389/fpsyt.2022.937330

**Published:** 2022-08-01

**Authors:** Pan Chen, Xiao-Hong Li, Zhaohui Su, Yi-Lang Tang, Yi Ma, Chee H. Ng, Yu-Tao Xiang

**Affiliations:** ^1^Unit of Psychiatry, Department of Public Health and Medicinal Administration, Institute of Translational Medicine, Faculty of Health Sciences, University of Macau, Macao, Macao SAR, China; ^2^Institute of Advanced Studies in Humanities and Social Sciences, University of Macau, Macao, Macao SAR, China; ^3^Centre for Cognitive and Brain Sciences, University of Macau, Macao, Macao SAR, China; ^4^The National Clinical Research Center for Mental Disorders, Beijing Key Laboratory of Mental Disorders, Beijing Anding Hospital, Capital Medical University, Beijing, China; ^5^School of Public Health, Southeast University, Nanjing, China; ^6^Department of Psychiatry and Behavioral Sciences, Emory University, Atlanta, GA, United States; ^7^Mental Health Service Line, Atlanta Veterans Affairs (VA) Medical Center, Decatur, GA, United States; ^8^Outpatient Department, Beijing Anzhen Hospital, Capital Medical University, Beijing, China; ^9^Department of Psychiatry, The Melbourne Clinic and St Vincent’s Hospital, University of Melbourne, Richmond, VIC, Australia

**Keywords:** schizophrenia, bibliometric analysis, reason, scientific misconduct, retracted publication

## Abstract

**Objectives:**

The growing rate of retraction of scientific publications has attracted much attention within the academic community, but there is little knowledge about the nature of such retractions in schizophrenia-related research. This study aimed to analyze the characteristics of retractions of schizophrenia-related publications.

**Materials and methods:**

The Web of Science was searched for eligible studies. A bibliometric analysis was conducted to describe the characteristics of the retractions using R software and Excel 2019. Content analysis was conducted to examine the essential components of retraction notices.

**Results:**

A total of 36 retracted publications with 415 citations were identified from 1997 to 2021, of which, 83.3% occurred in the last decade. The overall retraction rate was 0.19%, with most of them (29; 80.56%) from the United Kingdom. The retractions were published in 33 journals, and the 2020 IFs ranged between 0.17 and 49.96 (Median = 3.93). The retractions involved 21 research areas, particularly in Psychiatry (19; 52.78%), Neurosciences and Neurology (10; 27.78%), and Psychology (7; 19.44%). Data issues (17; 42.22%), administrative errors of the publishers (5; 13.89%), and study design (4; 11.11%) were the top three reasons for retractions.

**Conclusion:**

This study provides an insight into retractions of schizophrenia-related publications. Institutional governance should be further strengthened to improve the scrutiny of publications, prevent continuing citations, and erroneous propagation after retraction.

## Introduction

Retraction of peer-reviewed scientific publications is becoming more common. The growing rate of retractions with the increasing scientific publications in recent years has attracted much attention. Numerous reasons for retractions include concerns about data quality, and research misconduct, such as redundant publication, plagiarism, copyright infringement, unethical research, and peer review manipulation (1). The main objectives of retraction are to ensure research integrity rather than to punish authors, and to alert the academic community that these publications’ findings are not credible and should not be cited (2, 3). In the process of creating innovations, research outputs play a vital role as an important medium for information communication and presentation of findings. The filtering of these publications deserves attention, as the risk of disseminating inaccurate information and results of poor-quality research increases with the rapid expansion of ongoing research (4). For instance, to date, 196 articles related to COVID-19 have been retracted due to various reasons (5). If research articles are fraudulent, they could lead to catastrophic consequences on human health. In 1998, Dr. Andrew Wakefield and his colleagues unethically conducted research on children and concluded that there was a link between measles, mumps, rubella (MMR) vaccine and autism; consequently, thousands of parents refused MMR vaccines for their children which caused an outbreak of fatal measles (6). This misinformation continued to spread for at least 12 years, which had an immeasurable health impact on families with children with autism and also resulted in a huge financial cost to verify the accuracy of this finding (6). Another paper that was retracted from The Lancet due to doubtful data authenticity (7), claimed that hydroxychloroquine was ineffective in treating COVID-19 and even caused arrhythmias, which provoked a strong reaction in the scientific community and the public (8).

The retraction rate of scientific publications across many fields has been increasing in recent years. One study found a retraction rate of 0.38 per 10,000 publications in all fields in 1985, 2.03 in 2000, and 5.95 in 2014 (9), while another study found an average rate of 2.5 per 10,000 between 2013 and 2016 in all fields (10). A recent report found that the retraction rate has increased 10 times in veterinary medicine and animal health publications during the period between 1993 and 2019 (11). The trends of retractions varied by publication year (9) and research field (12, 13). The most common reason for retraction is academic misconduct, such as fraud or suspected fraud, duplicate publications, and plagiarism (13, 14).

Previous studies have reviewed the retracted literature in different specialties of medical fields, such as nursing and midwifery (15), neurosurgery (16), hematology (13), and anesthesiology (1). However, few studies have focused on the mental health literature (17). Schizophrenia is a severe mental disorder characterized by disturbances in perceived reality and behavior, such as persistent delusions, hallucinations, disorganized thinking, negative symptoms, and cognitive impairment (18–20). Approximately 1 in 300 people are affected worldwide, and its global disease burden has increased 11.4% from 1990 to 2019 (21, 22). Beyond the disease itself, relevant stigma and violations of human rights associated with schizophrenia can have a significant impact on the individual’s family, work, and social function (23–25). Additionally, schizophrenia patients have a shorter life expectancy when compared to the general population (18). Due to these features, schizophrenia is one of the most important areas of research in psychiatry and medicine.

A bibliometric analysis showed that the research on schizophrenia has been increasing in recent years, involving a wide range of research areas, including Psychiatry (69.8%), Neurosciences (20.7%), Clinical Neurology (12.4%), Pharmacology and Pharmacy (9.6%), and Genetics/Heredity (3.4%) (26). Schizophrenia related research has mainly focused on epidemiology, etiology, and treatment aspects, but the findings remained variable and at times controversial (18). Ensuring academic rigor is important for the medical advancement in schizophrenia and other fields of medicine. False research results could mislead the academic community or drive researchers in wrong directions, leading to much wasted research resources and negative impact on patient care and recovery (27, 28). Timely retraction is an important measure to prevent erroneous findings from being propagated. However, the nature of retractions in schizophrenia-related publications is unknown.

Bibliometric analysis has been widely used to examine the research trends in a specific field. Compared with traditional descriptive reviews of the literature, it is based on two components: one is the performance analysis that can provide the general characteristics of relevant publications, such as the number of publications, publication years, authors, institutions, countries, and journals; the other is science mapping, which examines and visualizes the relationships between research constituents such as co-citation analysis, bibliographic coupling, co-word and co-authorship analyses. In recent years, bibliometric analysis has been used to explore the characteristics of retractions in academic fields such as rehabilitation (29) and oncology (12). However, to the best of our knowledge, no bibliometric analysis on schizophrenia-related publication retractions has been published. As such, we performed a bibliometric analysis and content analysis of the characteristics of retracted publications on this topic, including publication years, sources, research areas, citations, and reasons for retractions.

## Materials and methods

The retracted publications on schizophrenia-related studies were searched from the Web of Science Core Collection in the database of the Web of Science (WoS). The search term was “TI/AB = *Schizophrenia OR Schizophrenias OR Schizoaffective OR Schizophreniform OR Psychosis OR Psychotic OR Schizophrenic Disorders OR Disorder, Schizophrenic OR Disorders, Schizophrenic OR Schizophrenic Disorder OR Schizophrenic Disorders OR Dementia Praecox OR Schizoaffective disorder OR Psychotic Disorders*.” The article type was limited into “*Retracted Publications and Retractions*.” A comprehensive screening procedure was conducted manually to avoid false positive or negative results.

The R software and Excel 2019 were used to summarize the characteristics of retractions, including the published year, countries, journals, and research areas. The Journal Impact Factor (IF) in 2020 was used for impact analysis of the journals. Given the rapid growth of journal impact factors, a 5-year IF was used to describe the impact of journals, as it is more stable compared to Immediacy IF. Furthermore, an IF without journal self-citation was used to reduce the potential inflation of IF because self-citation of journals could result in a higher IF value (30). In addition, content analysis was used to examine the reasons for retraction by reviewing the retraction notices. To analyze the transparency of retraction notices, this study included the following four essential components as previously recommended (3): initiators, cause, whether there was consensus between editors and authors on the retraction decisions, and whether retractions were related to the post-publication review (such as comments on PubPeer).

## Results

**FIGURE 1:**
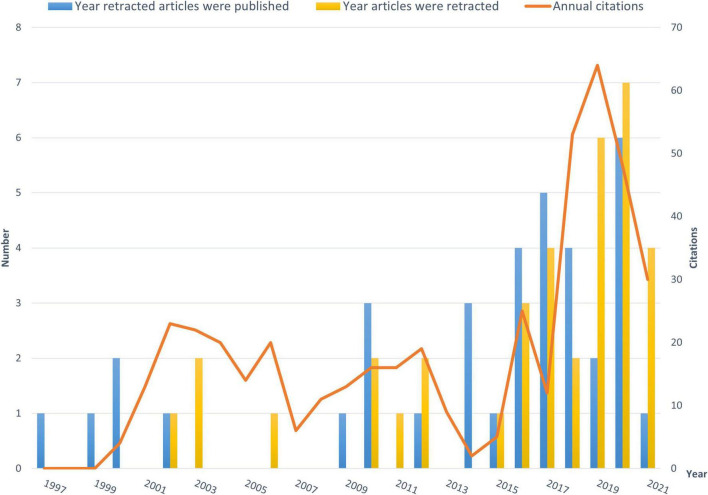
Distribution of retracted publications on schizophrenia-related studies during 1997 and 2021.

**FIGURE 2:**
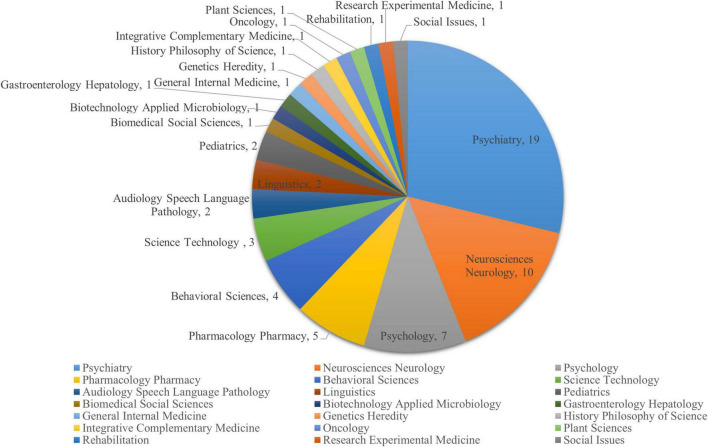
Research areas of retracted publications on schizophrenia-related studies.

A total of 19,176 publications on schizophrenia-related publications were searched in WoS from its inception to the search date (20/03/2022). Forty retractions were found after limiting the study types, four articles were discarded due to irrelevant topics or duplications in the process of data cleaning. Thirty-six publications from 1997 to 2021 were finally included in this study; of all the retractions, 83.3% (*N* = 30) occurred in the last decade. The overall retraction rate was 0.19%, and the years of retraction were between 2002 and 2021. The distribution of publication year, retraction year and annual citations are presented in Figure 1. The delay between publication and retraction time (year) ranged from 0 to 10 years with an average of 1.89 years (standard deviation = 2.22 years). The total citations of the 36 retracted publications were 415, with the most citations arising from a single publication (151) (31). The citations of the 36 retractions during the recent ten years accounted for 49.9% (207) of the total citations. Figure 2 presents the distribution of research areas of the retracted publications. The top three included Psychiatry (19, 52.78%), Neurosciences and Neurology (10, 27.78%), and Psychology (7, 19.44%).

The retracted publications were from 15 countries or regions. Table 1 shows the top five countries where the publications originated, including the United Kingdom (29; 80.56%), the United States (8; 22.22%), China (8; 22.22%), Canada (7; 19.44%), and Germany (7; 19.44%). Table 2 shows that the 36 retractions were published in 33 different journals. The IFs of the journals (in 2020) ranged between 0.17 and 49.96, with a median of 3.93. The Journal of Developmental and Behavioral Pediatrics (*IF* = 2.22), Journal of ECT (*IF* = 3.63), and Psychiatry and Clinical Neurosciences (*IF* = 5.12) each retracted two publications, respectively. A total of 17 journals (47.2%) were related to Psychology and Psychiatry.

**TABLE 1:** Countries/regions with retractions of schizophrenia-related studies.

Region	*N*	%*^a^*
The United Kingdom	29	80.56
The United States	8	22.22
China	8	22.22
Canada	7	19.44
Germany	7	19.44
Portugal	5	13.89
Spain	5	13.89
Iran	3	8.33
Japan	3	8.33
Sweden	3	8.33
India	2	5.56
Ireland	2	5.56
Armenia	1	2.78
Netherlands	1	2.78
Switzerland	1	2.78

N, number.

^a^Some publications involved more than one country; therefore, the total percentages were more than 100%.

**TABLE 2:** Journals with retracted publications on schizophrenia-related studies.

Journal	*N*	IF (2020)	IF (5 years)	IF without self-citations
Psychiatry and Clinical Neurosciences	2	5.19	4.80	4.92
Journal of ECT	2	3.64	3.01	3.29
Journal of Developmental and Behavioral Pediatrics	2	2.23	3.23	2.14
Nature	1	49.96	54.64	49.32
American Journal of Psychiatry	1	18.11	17.83	17.53
National Science Review	1	17.28	17.58	16.65
American Journal of Gastroenterology	1	10.86	12.59	10.43
British Journal of Psychiatry	1	9.31	10.24	9.10
Schizophrenia Bulletin	1	9.30	9.438	8.719
Alzheimer’s Research & Therapy	1	6.98	8.08	6.65
Translational Psychiatry	1	6.22	7.097	5.995
Biological Psychiatry-Cognitive Neuroscience and Neuroimaging	1	6.20	4.55	–
International Journal of Neuropsychopharmacology	1	5.18	5.17	5.03
Journal of Psychiatric Research	1	4.79	5.38	4.69
Journal of Clinical Psychiatry	1	4.38	5.40	4.15
Scientific Reports	1	4.38	5.13	4.17
Journal of Ethnopharmacology	1	4.36	4.49	3.97
British Journal of Clinical Psychology	1	4.13	4.33	3.93
Annals of Translational Medicine	1	3.93	4.63	3.47
Clinical Neurophysiology	1	3.71	4.57	3.24
Psychiatry Research	1	3.22	3.405	3.123
BJPsych Open	1	3.20	3.45	3.04
European Journal of Clinical Pharmacology	1	2.95	3.27	2.81
Neuropsychiatric Disease and Treatment	1	2.57	3.20	2.49
International Journal of Clinical Practice	1	2.50	2.726	2.404
Neuropsychobiology	1	2.33	2.30	2.31
New Genetics and Society	1	2.18	2.26	1.57
General Psychiatry	1	2.00	–	–
Human Psychopharmacology-Clinical and Experimental	1	1.67	2.81	1.61
Language and Speech	1	1.50	1.68	1.41
Clinical Linguistics & Phonetics	1	1.35	1.65	0.90
Actas Espanolas De Psiquiatria	1	1.20	2.07	1.13
Sante Mentale Au Quebec	1	0.17	0.24	–

IF, impact factor; “–”, not reported in Web of Science.

Supplementary Table 1 shows the characteristics of the retractions. All the 36 retraction notices reported the reasons for the retraction. In sum, nine (25.0%) retraction notices did not report who were the initiators, 24 (66.7%) did not report whether there was consensus between editors and authors on the retraction decisions, and 28 (77.8%) did not report whether retractions were related to the post-publication review. Table 3 lists the reasons for retraction as indicated by relevant journals, which include eight categories: data issues (17; 42.22%), administrative errors of the publisher (5; 13.89%), copyright (3; 8.33%), plagiarism (2; 5.56%), redundant publications (2; 5.56%), study design issues (4; 11.11%), ethical issues (1; 2.78%), and others (1, 2.78%). Based on the classification of reasons for paper retraction outlined by the Committee on Publication Ethics (COPE) (2), 11 (30.56%) out of the 36 retracted publications were due to honest errors, including occasional errors made in the original database or experimental data (7; 19.44%) and improper data manipulation (4; 11.11%).

**TABLE 3:** Reasons for retracted publications on schizophrenia-related studies.

Reason	Type	*N*	%
Data issues (17, 47.22%)	Honest error (Data error)	11	30.56
	Misconduct (Suspicious fabrication)	2	5.56
	Invalid data	3	8.33
	Unreplicable results	1	2.78
	No original data	1	2.78
Administrative errors of publisher	–	5	13.89
Study design (4, 11.11%)	Inconsistency with original study design	3	8.33
	Unclear methodology	1	2.78
Copyright	Material or data used without authorization	3	8.33
Plagiarism	–	2	5.56
Redundant publication	–	2	5.56
Ethic issue	No ethical approval	1	2.78
Others	No permission to publish by the author	1	2.78

## Discussion

This is the first bibliometric study on retractions of schizophrenia-related publications. We identified 36 retractions among 19,176 publications on schizophrenia-related studies. The overall retraction rate was relatively lower (0.19%) compared with other fields such as neurosurgery (7.3%) (16). Three-quarters of retractions occurred during the past decade (2011–2021), a trend similar to the retractions in the obstetrics literature where 76% of retractions occurred in the recent decade (2009–2019) (27). This is likely to be related to the overall growing number of academic publications, which may lead to increased academic errors. Additionally, publishers have promoted the awareness of scrutiny of publications (29). Academic misconduct has become a priority in the review procedure for many journals. Currently, many guidelines to standardize the process of retractions are available; of them, the most authoritative guidelines were issued by the COPE (2). The number of retractions has grown since the COPE guidelines were published in 2009 (29). Our analysis revealed that the average gap between publication and retraction time was 1.89 years, which is shorter than the retractions in other fields such as nursing and midwifery (2.3 years) (15) and life science research (3.8 years) (9). A longer delay in retraction may be associated with a more negative academic impact. The erroneous research findings may mislead other researchers, resulting in a waste of time, effort, and resources, and may even harm research participants (27). The high citations of retracted publications suggest that these retractions continued to have a certain impact on the schizophrenia research field as some were still cited even after retraction. Thus, a prompt retraction process is needed and clear signs and labels, such as attaching a clear watermark to the retraction (32), may be helpful in preventing further citations of retracted publications.

The retracted publications identified in this study involved 21 research areas; however, it should be noted that one retracted article may involve more than one research area. More than half of the retractions were classified in the field of Psychiatry (52.78%). The retractions also involved some experiment-based research areas such as Neurosciences, Neurology, Pharmacology, Pharmacy, and Behavioral Sciences. A previous study (29) found that publications based on basic experiments were more likely to be retracted for academic misconduct. Certain countries such as the United Kingdom, United States, China, Canada, and Germany were associated with the most retractions. However, these countries also contributed to the most publications in schizophrenia-related research; the United States contributed the most publications, followed by the United Kingdom, Germany, China, and Canada (26). Most retractions were published in journals related to Psychology/Psychiatry, such as the American Journal of Psychiatry, British Journal of Psychiatry, and Schizophrenia Bulletin. Given the small number of retractions per journal, we could not examine the correlation between the number of retractions and the impact factor of the journals involved. Previous studies on the relationship between retraction rate and journal impact factor found mixed results including positive (33), negative (15), and also non-significant associations (13).

This study analyzed all the retraction notices comprehensively to understand the degree of transparency of retractions. Incomplete information regarding the retraction notices will prevent any assessment of their historical and academic significance, while inadequate information can mislead or distort the readers and provide a biased view (34). Thus, promoting the transparency of the retraction notices is vital to maintain the scientific integrity by acting as a warning or discontinuation measure (35). Although the COPE released a guideline to formalize retraction notices, there has been little or no change to improve the transparency (3). In our study, although the reasons for schizophrenia-related retractions were reported, the other three essential components (e.g., initiators, whether there is consensus between editors and authors on the retraction decision, and whether retractions are related to the post-publication review) were mostly lacking. Possible reasons may include stigma (e.g., fear of reputational damage or legal responsibility), inconsistent requirements regarding the retraction notices between journals (34) and difficulties in implementation. Thus, reform in reporting retractions can encourage authors and publishers to explain the issues clearly and standardize the information provided among journals.

Overall, 86% of retractions of schizophrenia-related studies were attributed to author-related reasons. The most common reason was data issues, of which 64.7% were honest errors. For example, one paper published in Nature (111 citations) entitled “Microglia-dependent synapse loss in type I interferon-mediated lupus” was retracted due to the non-replicable results in the follow-up experiments (36). Another highly-cited paper entitled “Expression of Oct-6, a POUIII domain transcription factor, in schizophrenia” suggested that Oct-6 may be a marker of the neuropathology associated with schizophrenia (37). The data was suspected of being fraudulent; thus, the authenticity of this finding was in doubt. Other studies were retracted due to incomplete data (38), lack of original data (39), or errors in data processing that led to biased conclusions (40). These findings highlight the importance of data accuracy, integrity, and data double-checking.

In contrast, 13.89% of the retractions were due to journal or publisher reasons, such as administrative errors, suggesting publishers should enhance their measures to avoid such errors (16). Three retractions were due to inconsistencies between the contents or research methods and the original study design (41–43). For example, Ninomiya et al. (41) examined the long-term efficacy and safety of blonanserin for first-episode schizophrenia, which was retracted as subjects did not satisfy the inclusion criteria. Incorrect or inappropriate research results could mislead researchers, the public, or even entrepreneurs, resulting in wasted research funds, selection of ineffective drug treatments, and unethical profit-making (4, 44). A study may be invalid or potentially harmful if it does not align with the content of the original study registration. All clinical trials need to be registered before implementation, such as in the International Clinical Trials Registry Platform (ICTRP) supported by the World Health Organization (45). The aim is to ensure adequate knowledge about the research, increase research transparency, and strengthen the validity and value of the scientific evidence base (45).

The lack of ethical governance is another reason for retractions. One paper from the American Journal of Gastroenterology with 151 citations was retracted 10 years after publication; one of the reasons was having no local ethics committee approval (31). Thus, authors, editors and publishers should strengthen the consideration and review of all submitted research information including appropriate ethical approvals. The range and frequency of retraction reasons varied between different academic fields. For example, in both dentistry and obstetrics, redundant publications, and plagiarism were the most common reasons (27, 46), while in the field of pharmacy, falsification, or data manipulation were the most frequent (47), which are in contrast to our findings in schizophrenia-related research. The development and application of Plagiarism Detection software, such as iThenticate and Turnitin (16, 46), may contribute considerably to preventing plagiarism issues. Previous studies have proposed the notion of a “publish or perish” culture to explain the research misconduct issues (1, 9, 48). Quantity and quality of publications are associated with academic ranking, promotion and reputation; further monetary incentives in research commonly occur in some institutions/countries (49). Personality traits combined with highly competitive pressures appear to drive some researchers to falsify or fabricate data (1). Moreover, one study in China found that the majority of survey respondents considered that the current academic assessment system contributes heavily to academic misconduct and needs to be reformed to create a healthy academic environment (50). This supports the importance of developing appropriate academic assessment criteria for researchers. We suggest that the publication of good-quality research is a collaborative effort between organizations, publishers, journals and authors to ensure transparency in reporting, prevent research misconduct and disclose any research limitations.

There are several limitations to this study. First, following relevant guidelines of bibliometric analysis (51) and previous studies (52–54), the WoS was used in the literature search. However, the possibility that some studies may be missed could not be excluded. Second, some retraction notices were conservative in stating the reasons for retraction and the information was limited. For instance, where the study results were not reproducible, it was unclear whether this was due to research misconduct or honest errors. Thus, the retraction notices should be standardized to improve transparency. Third, not all academic misconduct could be uncovered by publishers or readers, therefore retraction rates may well be underestimated.

## Conclusion

This study provides an insight into retractions of schizophrenia-related research. The distribution of the retractions varied across countries, journals, and research areas. The number of annual retractions has risen over the past decade with the implementation of existing retraction guidelines, and honest errors account for most retractions. Transparency in reporting retraction notices should be implemented. Researchers should employ measures to ensure the authenticity of their research data. Institutional governance needs to improve the scrutiny of publications and prevent continuing citations and erroneous propagation after retraction.

## Data availability statement

The original contributions presented in this study are included in the article/Supplementary material, further inquiries can be directed to the corresponding authors.

## Author contributions

PC and Y-TX: study design. PC, X-HL, ZS, YM, and Y-TX: data collection, analysis, and interpretation. PC, Y-LT, and Y-TX: drafting of the manuscript. CN: critical revision of the manuscript. All authors approval of the final version for publication.
