# Supplementary material for: Characteristics of global retractions of schizophrenia-related publications: A bibliometric analysis
Source: Front Psychiatry. 2022 Aug 1;13:937330. doi: 10.3389/fpsyt.2022.937330 (PMC9376617; doi:10.3389/fpsyt.2022.937330)
Supplement: Supplementary file 1 [file Table_1.docx]

**Table S1. The characteristics of retractions**

| **No.** | **First author** | **Title** | **Journal** | **Publication year** | **Retraction year** | **Total citations** | **Components of retraction notices** | | | |
| --- | --- | --- | --- | --- | --- | --- | --- | --- | --- | --- |
|  |  |  |  |  |  |  | **Reason** | **Initiators** | **Whether there is consensus between editors and authors about retractions** | **Whether post-publication review** |
| 1 | ABU-AKEL A, et al | A study of cohesive patterns and dynamic choices utilized by two schizophrenic patients in dialog, pre- and post-medication | Language and Speech | 1997 | 2003 | 6 | Redundant publication | Publisher | NR | NR |
| 2 | ABU-AKEL A, et al | Phoricity as a measure of Clozaril's efficacy in treating disorganized schizophrenia | Clinical Linguistics & Phonetics | 1999 | 2003 | 1 | Plagiarism | Editor | NR | NR |
| 3 | BEAUDOIN M, et al. | Characterization of First Episodes of Schizophrenia from Combined Administrative Databases | Sante Mentale Au Quebec | 2018 | 2019 | 1 | Honest error (Data error) | Author | NR | NR |
| 4 | BHUI K, et al | Trauma, khat and common psychotic symptoms: A quantitative study | Journal of Ethnopharmacology | 2010 | 2011 | 3 | Administrative errors of publisher | Author | NR | NR |
| 5 | BIALAS AR, et al. | Microglia-dependent synapse loss in type I interferon-mediated lupus | Nature | 2017 | 2020 | 111 | Unrepeatable results | Author | NR | NR |
| 6 | BROHL H, et al. | Neural correlates of psychopathological symptoms in patients with affective and psychotic disorders | Clinical Neurophysiology | 2017 | 2018 | 0 | No permission to publish by the author) | Publisher | NR | NR |
| 7 | DAVIS KAS, et al. | Mental health in UK Biobank: development, implementation and results from an online questionnaire completed by 157 366 participants | BJPsych Open | 2018 | 2019 | 47 | Honest error (Data error) | Editor | Yes | NR |
| 8 | FEIFEL D, et al. | Safety and tolerability of a rapidly escalating dose-loading regimen for risperidone | Journal of Clinical Psychiatry | 2000 | 2002 | 9 | Unclear methodology | Author | NR | NR |
| 9 | GHADERI A, et al. | Effect of N-acetyl cysteine (NAC) supplementation on positive and negative syndrome scale in schizophrenia: a systematic review and meta-analysis of randomised controlled trials | European Journal of Clinical Pharmacology | 2019 | 2019 | 0 | Honest error (Data error) | Author | NR | NR |
| 10 | GRAVE J, et al. | The effects of perceptual load in processing emotional facial expression in psychotic disorders | Psychiatry Research | 2017 | 2021 | 0 | Honest error (Data error) | Author and Editor | Yes | NR |
| 11 | GULBOL S, et al. | Withdrawal: "Determination of depression, burnout and psychological resilience levels of caregivers of schizophrenia patients" Selin Gulbol, Mustafa Ari, Mehmet Hanifi Kokacya | International Journal of Clinical Practice | 2021 | 2021 | 1 | Administrative errors of publisher | NR | NR | NR |
| 12 | GUMLEY A, et al. | A meta-analysis and theoretical critique of oxytocin and psychosis: Prospects for attachment and compassion in promoting recovery | British Journal of Clinical Psychology | 2014 | 2017 | 0 | Honest error (Data error) | NR | Yes | No |
| 13 | GUO ZH, et al. | Brief cognitive-behavioural therapy for patients in the community with schizophrenia: Randomised controlled trial in Beijing, China | British Journal of Psychiatry | 2017 | 2019 | 1 | Inconsistency with original study design | Editor | Yes | Yes |
| 14 | ILIA M, et al. | Expression of Oct-6, a POUIII domain transcription factor, in schizophrenia | American Journal of Psychiatry | 2002 | 2006 | 18 | Misconduct (Suspicious fabrication) | Author | NR | NR |
| 15 | INOSHITA M, et al. | A significant causal association between C-reactive protein levels and schizophrenia | Scientific Reports | 2016 | 2018 | 6 | Honest error (Data error) | Author | NR | NR |
| 16 | JANARDHANAN A, et al. | Nardostachys jatamansi Targets BDNF-TrkB to Alleviate Ketamine-Induced Schizophrenia-Like Symptoms in Rats | Neuropsychobiology | 2016 | 2021 | 9 | Misconduct (Suspicious fabrication) | Editor | No | Yes |
| 17 | JIANG S, et al. | Identification of de novo mutations in prenatal neurodevelopment-associated genes in schizophrenia in two Han Chinese patient-sibling family-based cohorts | Translational Psychiatry | 2020 | 2020 | 0 | Material or data used without authorization | Author | NR | NR |
| 18 | KHODAYAR-PARDO P, et al. | Sudden Complex Hallucinations in a 14-Year-Old Girl: Schizophrenia Spectrum Disorders versus Dissociative Disorders-The Influence of Early Life Experiences on Future Mental Health | Journal of Developmental and Behavioral Pediatrics | 2020 | 2020 | 2 | Administrative error | Publisher | NR | NR |
| 19 | KHODAYAR-PARDO P, et al. | Abrupt and Severe Obsessive-Compulsive Disorder in an 11-Year-Old Girl-PANDAS/PANS Syndrome: An Entity to be Considered-Management Implications | Journal of Developmental and Behavioral Pediatrics | 2020 | 2020 | 2 | Administrative error | Publisher | NR | NR |
| 20 | KILCIKSIZ, NA, et al. | M111. Quantitative Assessment of Mania and Psychosis During Hospitalization Using Automated Analysis of Face, Voice, and Language | Schizophrenia Bulletin | 2020 | 2020 | 0 | Material or data used without authorization | NR | NR | NR |
| 21 | KRAGULJAC NV, et al. | Mnemonic Discrimination Deficits in First-Episode Psychosis and a Ketamine Model Suggests Dentate Gyrus Pathology Linked to N-Methyl-D-Aspartate Receptor Hypofunction | Biological Psychiatry-Cognitive Neuroscience and Neuroimaging | 2018 | 2021 | 0 | Honest error (Data error) | Author | Yes | NR |
| 22 | LI M, 2017, et al. | Illuminating the dark road from schizophrenia genetic associations to disease mechanisms | National Science Review | 2017 | 2017 | 0 | Plagiarism | Author | NR | NR |
| 23 | NIKISCH G, et al. | Relationship between dopamine D-2 receptor occupancy, clinical response, and drug and monoamine metabolites levels in plasma and cerebrospinal fluid. A pilot study in patients suffering from first-episode schizophrenia treated with quetiapine | Journal of Psychiatric Research | 2010 | 2012 | 14 | Invalid data | Author | Yes | NR |
| 24 | NINOMIYA Y, et al. | Long-term efficacy and safety of blonanserin in patients with first-episode schizophrenia: A 1-year open-label trial | Psychiatry and Clinical Neurosciences | 2014 | 2017 | 7 | Inconsistency with original study design | NR | Yes | Yes |
| 25 | PRIYA I, et al. | A review of 45 candidate genes: association of single nucleotide polymorphism to schizophrenia risk | New Genetics and Society | 2018 | 2019 | 0 | Administrative error | Publisher | NR | NR |
| 26 | REN ZB, et al. | A cross-sectional study on perception of stigma by Chinese schizophrenia patients | Neuropsychiatric Disease and Treatment | 2014 | 2015 | 3 | No original data | NR | NR | NR |
| 27 | TAJIMA K, et al. | Schizophrenia treatment. Critical review on the drugs and mechanisms of action of antipsychotics | Actas Espanolas De Psiquiatria | 2009 | 2012 | 18 | Duplicate publication | Editor | NR | NR |
| 28 | TAN L, et al. | Efficacy and safety of atypical antipsychotic drug treatment for dementia: a systematic review and meta-analysis | Alzheimers Research & Therapy | 2015 | 2016 | 33 | Honest error (Data error) | Author | NR | NR |
| 29 | TAN Y, et al. | Elevated serum S100B protein in the first-episode drug-naive patients with schizophrenia in Chinese population | International Journal of Neuropsychopharmacology | 2010 | 2010 | 0 | Invalid data | Author and Editor | Yes | NR |
| 30 | TENJIN T, et al. | Effect of blonanserin on cognitive function in antipsychotic-naive first-episode schizophrenia | Human Psychopharmacology-Clinical and Experimental | 2012 | 2017 | 0 | Inconsistency with original study design | NR | Yes | Yes |
| 31 | USUKI M, et al. | Retraction statement: National database study on the use of long-acting antipsychotic injections and hospital readmission proportions in patients with schizophrenia in Japan. | Psychiatry and Clinical Neurosciences | 2020 | 2020 | 0 | Material or data used without authorization | NR | Yes | Yes |
| 32 | WAKEFIELD AJ, et al.L | Enterocolitis in children with developmental disorders | American Journal of Gastroenterology | 2000 | 2010 | 151 | No ethical approval | Editor | NR | NR |
| 33 | WANG W, et al. | Cognitive-behavioural therapy for personal recovery of patients with schizophrenia: a systematic review and meta-analysis | General Psychiatry | 2019 | 2019 | 0 | Honest error (Data error) | NR | Yes | NR |
| 34 | YANG H, et al. | Methylation of the MAOA promoter is associated with schizophrenia | Annals of Translational Medicine | 2020 | 2020 | 0 | Invalid data | NR | NR | NR |
| 35 | ZHENG W, et al.(a) | Electroconvulsive Therapy Alone for Schizophrenia: A Meta-analysis of Randomized, Single-blind, Controlled Trials | Journal of Ect | 2016 | 2016 | 1 | Honest error (Data error) | Editor and Publisher | NR | Yes |
| 36 | ZHENG W, et al.(b) | Adjunctive Electroconvulsive Therapy for Schizophrenia: A Meta- analysis of Randomized Rater- Masked Controlled Trials | Journal of Ect | 2016 | 2016 | 1 | Honest error (Data error) | Editor and Publisher | NR | Yes |

NR: Not reported
